# Changes in Phenolics and Fatty Acids Composition and Related Gene Expression during the Development from Seed to Leaves of Three Cultivated Cardoon Genotypes

**DOI:** 10.3390/antiox9111096

**Published:** 2020-11-08

**Authors:** Giulia Graziani, Teresa Docimo, Monica De Palma, Francesca Sparvoli, Luana Izzo, Marina Tucci, Alberto Ritieni

**Affiliations:** 1 Department of Pharmacy, University of Naples Federico II, Via Domenico Montesano 49, 80131 Naples, Italy; luana.izzo@unina.it (L.I.); ritialb@unina.it (A.R.); 2 Institute of Bioscience and Bioresources, Consiglio Nazionale delle Ricerche, via Università 133, 80055 Portici, Italy; teresa.docimo@ibbr.cnr.it (T.D.); monica.depalma@ibbr.cnr.it (M.D.P.); 3 Institute of Agricultural Biology and Biotechnology, Consiglio Nazionale delle Ricerche, Via E. Bassini 15, 20133 Milan, Italy; sparvoli@ibba.cnr.it; 4 Unesco Chair for Health Education and Sustainable Development, 80131 Naples, Italy

**Keywords:** cardoon, multipurpose plant, chlorogenic acid, fatty acids, antioxidant

## Abstract

Cultivated cardoon (*Cynara cardunculus* var. *altilis*) has long been used as a food and medicine remedy and nowadays is considered a functional food. Its leaf bioactive compounds are mostly represented by chlorogenic acids and coumaroyl derivatives, known for their nutritional value and bioactivity. Having antioxidant and hepatoprotective properties, these molecules are used for medicinal purposes. Apart from the phenolic compounds in green tissues, cultivated cardoon is also used for the seed oil, having a composition suitable for the human diet, but also valuable as feedstock for the production of biofuel and biodegradable bioplastics. Given the wide spectrum of valuable cardoon molecules and their numerous industrial applications, a detailed characterization of different organs and tissues for their metabolic profiles as well as an extensive transcriptional analysis of associated key biosynthetic genes were performed to provide a deeper insight into metabolites biosynthesis and accumulation sites. This study aimed to provide a comprehensive analysis of the phenylpropanoids profile through UHPLC-Q-Orbitrap HRMS analysis, of fatty acids content through GC-MS analysis, along with quantitative transcriptional analyses by qRT-PCR of hydroxycinnamoyl-quinate transferase (*HQT*), stearic acid desaturase (*SAD*), and fatty acid desaturase (*FAD*) genes in seeds, hypocotyls, cotyledons and leaves of the cardoon genotypes “Spagnolo”, “Bianco Avorio”, and “Gigante”. Both oil yield and total phenols accumulation in all the tissues and organs indicated higher production in “Bianco Avorio” and “Spagnolo” than in “Gigante”. Antioxidant activity evaluation by DPPH, ABTS, and FRAP assays mirrored total phenols content. Overall, this study provides a detailed analysis of tissue composition of cardoon, enabling to elucidate value-added product accumulation and distribution during plant development and hence contributing to better address and optimize the sustainable use of this natural resource. Besides, our metabolic and transcriptional screening could be useful to guide the selection of superior genotypes.

## 1. Introduction

*Cynara cardunculus* L. var. *altilis* DC., the cultivated cardoon, belongs to the Asteraceae family and, with its sister species globe artichoke (*Cynara cardunculus* var. *scolymus*) and their common ancestor wild cardoon (*Cynara cardunculus* var. *sylvestris*), participates in the small *Cynara* genus. Cardoon and artichoke originate from the Mediterranean area [1], where they are mostly cultivated. Artichoke is famous as a food crop for its edible immature inflorescences (heads) and is also produced in America and Asia, while cardoon, although having a similar composition, has a restricted traditional food use in Mediterranean countries, though its cultivation for industrial purposes is increasing. Indeed, cultivated cardoon is gaining interest as a multipurpose crop, due to the versatility of its main products, which offer a wide spectrum of applications [2]. Due to its high biomass and related content of cellulose, emicellulose, and lignin, cardoon is starting to be exploited for energy production and green chemistry [3,4,5,6]. Concomitantly, cardoon extracts maintain a prominent role in the nutraceutical and pharmaceutical sectors, since numerous bioactive molecules are biosynthesized and accumulated in different parts of the plant. In particular, most of the health-beneficial compounds are polyphenols, which are present in all plant organs, though in higher amounts in leaves and seeds [7].

Cardoon biosynthesizes specialized metabolites, which are distinctive of Asteraceae [8], namely flavonoids, such as apigenin and luteolin, and hydroxycinnamic derivatives such as mono- and di-caffeoylquinic acids (CGA) [9,10,11]. Extraction of these cardoon bioactive compounds has been reported in many works [7,12,13] and several in vitro and in vivo studies have demonstrated the health-promoting effects related to its polyphenols. Among these polyphenols, chlorogenic acid (CGA) represents the major bioactive component because of its abundance and numerous bioactive functions, especially as a free-radical scavenger and antioxidant. These functions seem to be associated with a well-known dual role as a protectant against oxidative injury caused by free radicals [14,15] and as a substrate for both chemical and enzymatic browning reactions [9]. Apart from CGA, most cardoon phenolics confer several medicinal properties to cardoon leaf extracts, such as cholesterol-lowering, hepatoprotective, and diuretic activities, but also antifungal and antibacterial properties [16,17,18,19,20]. The biosynthesis of general phenylpropanoids and CGA proceeds through several sequential enzymatic reactions, starting from the amino-acid phenylalanine that, being the substrate of phenylalanine-ammonia-lyase (PAL), is the entry point for the formation of a plethora of phenylpropanoids. Afterward, cinnamate 4-hydroxylase (C4H) and 4-coumarate-CoA ligase generate *p*-coumaroyl-CoA, which is the precursor of both CGA and flavonoids. Three possible routes of CGA biosynthesis have been proposed in several plant species [21,22]. The route characterized in artichoke and other Asteraceae [23,24] proceeds through the catalytic activity of hydroxycinnamoyl-CoA quinate transferase (*HQT*) on caffeoyl-CoA and quinic acid to form CGA. Several *HQT* genes with catalytic activity towards *p*-coumaroyl-CoA have been characterized in Asteraceae species, and it cannot be excluded that several other *HQT* isoforms with a possible tissue-specific activity and specific regulation [25] are still undiscovered. In this regard, the study of the spatio-temporal expression pattern of *HQT* along with dynamic changes in CGA accumulation and distribution can provide insights into possible biosynthetic correlations and any developmental regulatory factors acting on the accumulation of this specialized metabolite. 

Indeed, cardoon use is not limited to phenylpropanoids, but also oils. As oleaginous species, cardoon produces seed oils, which have been investigated both for human nutrition [26] and as a potential renewable source for the production of biofuels and bioplastics [27]. In plants, fatty acids and derived molecules represent an energy source but are also fundamental in many metabolic and signaling processes, other than being structural components of membranes and thus directly involved in defenses responses [28,29]. In human and animal nutrition, fatty acids such as oleic and linoleic are essential major nutrients since mammals cannot synthesize but only uptake them through the diet. In plants, fatty acids are synthesized in the plastids starting from acetyl-CoA, and, after passing into the cytosol, are modified and assembled in lipids in the endoplasmic reticulum (ER) [30,31]. The desaturation of stearic acid (C18:0) to oleic acid (C18:1) is catalyzed by stearoyl-acyl carrier protein desaturase (SAD). Further desaturation of oleic to linoleic acid (18:2) is catalyzed by FAD2 in the ER and FAD6 in the plastid [32]. As oleic (C18:1Δ9) and linoleic acids (C18:2Δ9,12) are the main determinants for oil quality, their content is very important to assess the nutritional properties of edible oils or direct their technological applications [33,34]. Oleic and linoleic acids have both the capability of lowering total serum cholesterols, but oleic acid, having one double bond less than linoleic, has higher oxidative stability. For this reason, high oleic acid content has also a great potential for industrial uses, for example for making products such as biodiesel and lubricants, needing high oxidative stability. In this regard, the knowledge of active genes in oleic and linoleic biosynthesis is important for selection of MUFA- (mono-unsaturated fatty acids) and/or PUFA- (poly unsaturated fatty acids) enriched tissues and genotypes.

In our study, we followed the distribution and accumulation of phenylpropanoids and oils during the growth and development of cultivated cardoon from seeds to leaf formation. We characterized the metabolic composition of seeds, hypocotyls, cotyledons, and leaves of the three cultivated cardoon genotypes “Bianco Avorio”, “Spagnolo”, and “Gigante” by GC and high-resolution mass spectrometric analysis, along with the tissue and developmental expression of key genes in the biosynthesis of chlorogenic acid and oleic and linoleic acid by qRT-PCR. To minimize the effects of environment and growth conditions, that are known to influence the level and composition of these metabolites [35], we grew the three cardoon genotypes in controlled greenhouse conditions, thus allowing to specifically highlight genotype differences and assist the selection of the most suitable tissues and genotypes for different purposes. Moreover, to characterize tissues and genotypes based on the biological properties, we analyzed the antioxidant activities by ABTS, DPPH, and FRAP for all the tissues.

## 2. Materials and Methods

### 2.1. Reagents and Materials

Polyphenolic standards (purity > 98%), including luteolin, apigenin, diosmin, apigenin-8-C-glucoside, naringenin, quercetin-3-*O*-glucoside, quercetin, kaempferol, myricetin, naringin, kaempferol-3-*O*-glucoside, luteolin-7-*O*-glucoside, *p*-coumaric acid, chlorogenic acid, and 4-hydroxybenzoic acid were purchased from Sigma-Aldrich Chemical Co. (St. Louis, MO, USA). The reference standards were accurately weighed and dissolved in methanol to prepare stock solutions (1 mg mL^−1^). For the construction of calibration curves, a multi-compound working solution was prepared by appropriate dilution of stock solutions with methanol–water (80:20 *v/v*) to make concentrations in the range 0.02–5 µg·mL^−1^. For the antioxidant tests, gallic acid, 6-hydroxy-2,5,7,8-tetramethylchromane-2-carboxylic acid (Trolox), 1,1-diphenyl-2-picrylhydrazyl (DPPH), 2,3,5-triphenyltetrazolium chloride (TPTZ), anhydrous ferric chloride, hydrochloric acid, potassium persulfate (K_2_S_2_O_8_), 2,2’-azino-bis(3-ethylbenzothiazoline-6-sulfonic acid) diammonium salt (ABTS) and sodium acetate were purchased from Sigma-Aldrich (MO, United States). Methanol (MeOH), ethanol, and water (LC-MS grade) were acquired from Carlo Erba reagents (Milan, Italy), whereas formic acid (98–100%) was purchased from Fluka (Milan, Italy).

### 2.2. Plant Growth and Sample Collection

Seeds of *C. cardunculus* L. var. *altilis* DC. “Gigante” and “Spagnolo” genotypes were provided by ARCA 2010 s.c.a.r.l., Acerra (Italy), and “Bianco Avorio” seeds were provided by La Semiorto Sementi, Lavorate di Sarno (Italy). Seeds were placed in germination pots in the greenhouse of the Institute of Bioscience and Bioresources, Portici, (Italy), and grown for the collection of the different tissues at different vegetative stages (Supplementary Figure S1). Part of the germinated seeds were used for the collection of hypocotyls and cotyledons. Hypocotyls were cut with a razor blade, approx. 5 mm below the cotyledons, about 7–10 days post-germination. Other germinated seeds were used for the collection of cotyledons, and leaves were collected 30 days after emergence. Seeds, hypocotyls, cotyledons, and leaves were flash frozen at −80 °C and stored for RNA extraction and biochemical characterization.

### 2.3. RNA Extraction and qRT-PCR

Seeds and tissues of cultivated cardoon were harvested in biological triplicates and ground in liquid nitrogen. Total RNA was extracted from 100 mg of tissue using the RNeasy kit (Qiagen, CA, USA). After verification of RNA integrity and NanoDrop ND-8000 spectrophotometer (Fisher Scientific, DE, USA) quantification, 1 μg of total RNA was reversed transcribed using the QuantiTec Reverse Transcription Kit (Quiagen, CA, USA), according to the manufacturer’s instructions. Real-time qRT-PCR was performed with Platinum^®^ SYBR^®^ Green qPCR SuperMix (Applied Biosystem, CA, USA) in an ABI7900 HT (Life Technologies, CA, USA). Each PCR reaction (20 μL) contained 10 μL real-time qRT-PCR Mix, 4 μL of a 1:25 dilution of cDNA, and 0.25 μM of each specific primer designed on sequences retrieved from NCBI. Thermal cycling conditions were as follows: 50 °C for 2 min, 95 °C for 2 min, followed by 40 cycles of 15 s at 95 °C and 30 s at 60 °C. The dissociation stage was performed for each primer pairs and a single melting curve for each analyzed gene indicated the amplification of a single band. All reactions were performed on biological triplicates and technical duplicates and the 2^−ΔΔCT^ method [36,37] was used for fold change measurements. For the relative expression of each gene in each genotype, the tissue with the lowest expression level was used as an internal calibrator by setting its expression equal to 1. *C. cardunculus* actin gene, as previously reported [38] was used to normalize gene expression. In Supplementary Table S1, the accession number and sequences of primers used for real-time qRT-PCR are reported.

### 2.4. Ultrasound-Assisted Extraction of Polyphenolic Compounds

Lyophilized samples were extracted using the method reported in the literature [39] with few modifications. In particular, 3 g of dried sample were extracted with 30 mL of ethanol/water (50:50 *v/v*) by sonication at room temperature for 30 min. Samples were centrifuged to 4000 rpm at 4 °C, filtered through 0.45 mm nylon syringe membranes, and then used for high-resolution mass spectrometry analysis and antioxidant activity assay. The ultrasonic plant material extraction procedure was repeated three times.

### 2.5. Antioxidant Activity: ABTS Assay

The antioxidant capacity assay was conducted based on the method described by [40]. Briefly, 44 µL of aqueous 2.45 mM potassium persulfate were added to aqueous 7mM ABTS and incubated in the dark at room temperature (23 °C) for 12–16 h. After dilution (1:88) with ethanol, the absorbance of this ABTS^+^ working solution should be 0.700 ± 0.050 at 734 nm. The assay was performed by adding 0.1 mL of filtered and suitably diluted sample to 1 mL of ABTS^+^ working solution and the absorbance was monitored at 734 nm after 2.5 min. Results were expressed as Trolox^®^ equivalent antioxidant capacity (TEAC, mmol Trolox^®^ equivalents Kg^-1^ dry weight of plant). All determinations were performed in triplicate.

### 2.6. Antioxidant Activity: DPPH Assay

The DPPH radical-scavenging activity was determined using the method proposed by [41] with minor modifications. Briefly, the DPPH methanolic solution 100 μM (1 mL) was added to polyphenol extract (0.2 mL), and the decrease in absorbance of the resulting solution was monitored at 517 nm after 10 min. The results were corrected for dilution and expressed as Trolox^®^ equivalent antioxidant capacity (TEAC, mmol Trolox^®^ equivalents Kg^−1^ dry weight of plant). All determinations were performed in triplicate.

### 2.7. Antioxidant Activity: FRAP Assay

The FRAP assay was conducted according to the method reported by [42] with slight adaptations. Briefly, the FRAP reagent contained a solution of 10 μM TPTZ in 40 μM HCl, 20 μM of aqueous FeCl_3_ and acetate buffer (300 μM, pH 3.6) at 1:1:10 (*v/v/v*). The FRAP reagent (300 μL) and sample solutions (10 μL) were mixed and the absorbance was monitored at 593 nm after 10 min. The results were expressed in mmol Trolox^®^ Kg^−1^ dry weight (dw). The results were corrected for dilution and expressed as Trolox^®^ equivalent antioxidant capacity (TEAC, mmol Trolox^®^ equivalents Kg^−1^ dry weight of plant). All determinations were performed in triplicate.

### 2.8. HRMS Orbitrap Analysis of Bioactive Polyphenols

An Ultra-High-Pressure Liquid Chromatograph (UHPLC, Dionex UltiMate 3000, Thermo Fisher Scientific, Ma, USA) coupled with a Q-Exactive Orbitrap mass spectrometer (UHPLC, Thermo Fischer Scientific, Ma, USA) was used to investigate the quali-quantitative profile of polyphenolic compounds applying conditions reported in our previous work [43]. A Kinetex 2.6 µm Biphenyl (100 × 2.1 mm, Phenomenex) column was applied for chromatographic separation of polyphenols with a column temperature set at 25 °C. The mobile phase consisted of water containing 0.1% of formic acid (eluent A) and methanol containing 0.1% of formic acid (eluent B). Polyphenolic compounds were eluted using the following gradient program: 0–1.3 min 5% B, 1.3–9.3 min 5–100% B, 9.3–11.3 min 100% B, 11.3–13.3 min 100–5% B, 13.3–20 min 5% B. The flow rate was 0.2 mL min^−1^ and the injection volume was 2 μL. The mass spectrometer was operated in negative ion mode (ESI^-^) setting two scan events (Full ion MS and All ion fragmentation, AIF) for all compounds of interest. Full scan data were acquired setting a resolving power of 35,000 FWHM (full width at half maximum) at *m/z* 200. The key parameters were as follows: spray voltage −2.8 kV, sheath gas flow rate 35 arbitrary units, auxiliary-gas flow rate, 10 arbitrary units, capillary temperature 310 °C, auxiliary gas heater temperature 350 °C, S-lens RF level 50. For the scan event of AIF, the resolving power was set at 17,500 FWHM, the collision energies were 10, 20, and 45 eV, and the scan range was *m/z* 80–1200. Data acquisition and processing were performed with Quan/Qual Browser Xcalibur software, v. 3.1.66.10 (Xcalibur, Thermo Fisher Scientific, (Thermo Fisher Scientific, Waltham, MA, USA). Parameters of UHPLC-HRMS Orbitrap validation are reported in Supplementary Table S2.

### 2.9. Oil Extraction

The oil from the samples was extracted using solvent extraction according to the procedure described in the literature [44]. In particular, 100 mL of hexane were added to 10 g of powdered sample and shaken in a conical flask for 24 h. Then the solution was filtered under reduced pressure through filter paper (Whatman No. 1) and concentrated to remove completely hexane on a rotary evaporator (Rotavapor RE 120; Büchi, Flavil, Sweden). The final weight of solid material left after evaporation was recorded and used for the fatty acid profile study.

### 2.10. Fatty Acids Analysis

Preparation of fatty acid methyl ester derivatives (FAMEs) was carried out dissolving 0.1 g of oil sample in 1 mL hexane and 500 μL of methanolic KOH (2N). Afterward, the mixture was vortexed for 2 min at room temperature and the upper layer (hexane) was directly injected into GCMS. The fatty acid composition was investigated by GC-MS (Agilent Technologies, Santa Clara, CA, USA) using a capillary column, Rxi-5MS 5% Phenyl 95% Dimethylpolysiloxane (29.7 m × 0.25 mm i.d., 0.25 µm) (Restek, Bellefonte, PA, USA). One µL of the sample was injected in splitless mode into the injection port. Helium (grade 6.0) was used as carrier gas with a flow rate of 1.4 mL min^−1^. The temperature program of the column was as follows: the initial oven temperature = 50 °C for 1.5 min then raising from 50 °C to 80 °C with a program ramp rate of 2.5 °C/min, the column was kept at 80 °C for 1 min then up to 300 °C at 10 °C min^−1^, making a total run time of 36 min. The injector temperature was 280 °C. 

The mass spectrometer was tuned according to the manufacturer’s recommendation using perfluorotributylamine (PFTBA). The raw GC- TOF-MS data were processed by the ChromaTOF software version 5.03.09.0 (LECO Corporation, Saint Joseph, MI, USA) that deconvolutes mass spectra and performs peak identification using the NIST11 library (NIST, MD, USA). Results were expressed as mass response areas in relative percentages.

### 2.11. Statistical Analysis

Statistical analyses were performed using SigmaStat version 11.0 software (Sigma Stat, Statcon, Witzenhausen, Germany, SigmaStat for Windows). Mean and standard deviation were calculated on three biological and two technical replicates for all the biochemical and qRT-PCR data. Statistical differences were evaluated through a one-way analysis of variance (ANOVA). Tukey’s post hoc test was used for mean separation and the statistical significance of the comparisons was defined as *p* < 0.05.

## 3. Results and Discussion

### 3.1. Polyphenol Profiling of Cardoon Genotypes

Qualitative and quantitative profiles of polyphenolic compounds were obtained using an ultra-high-performance liquid chromatography method coupled with electrospray ionization hybrid linear trap quadrupole orbitrap mass spectrometry (UHPLC-Q-Orbitrap-HRMS). Data were analyzed using Qual Browser Xcalibur 3.0 (Thermo Fisher Scientific), and identification of individual compounds was supported by retention times, exact mass spectra data, and MS/MS spectra. Individual phenolic compounds were quantified using the calibration curves of the respective reference compounds as described in Section 2.1.

The bibliographic data indicated that the chemical profile of cardoon depends on the variety [45,46] and geographical origin [47]. The UHPLC technique coupled to Orbitrap-HRMS showed that the different analyzed cardoon tissues and organs contained the same phenolic qualitative profile, but their relative abundances were considerably different between samples and genotypes. A typical full-scan MS chromatogram of a cardoon sample (cotyledons) is reported in Supplementary Figure S2. The studied cardoon parts, namely seeds, hypocotyls, cotyledons, and leaves, showed significant differences in phenolic compounds content, with leaves having the highest total phenolic content and reaching an average value of 3.99 mg g^−1^ on a dry weight (dw) basis. Table 1 summarizes the peak characteristics of fifteen detected phenolic compounds. Two phenolic acids (peaks 1 and 3), nine non-anthocyanin flavonoids (peaks 4, 5, 6, 7, 8, 9, 10, 11, 12, 13, 14, and 15), and one caffeoylquinic acid (peak 2) were identified in *C. cardunculus* samples.

Flavonoids are represented by the greatest number of compounds, among which the most abundant were apigenin-8-C-glucoside (vitexin), luteolin glucoside, naringin, and luteolin, which is in accordance with the review by [48]. Peak 1 and 3 were positively identified as 4-hydroxy benzoic and coumaric acid, respectively, by comparing their retention time and mass characteristics with commercial standards. Regarding peak 2, caffeoylquinic acid, ([M − H]^−^ at *m/z* 353) was positively identified as 5-O-caffeoylquinic acid, yielding the base peak at *m/z* 191 and a secondary ion at *m/z* 179, characteristic of 5-acylchlorogenic acids, as reported by [49]. Peak 4 ([M − H]^−^ at *m/z* 463) released a unique MS^2^ unit at *m/z* 301 (−162 μ), corresponding to the loss of a glucosyl unit, being tentatively identified as quercetin 3-*O*-glucoside. Peak 5 was identified as apigenin 8-*C*-glucoside, presenting pseudomolecular ion [M − H]^−^ at *m/z* 431 and an MS^2^ fragment at *m/z* 269, corresponding to the loss of glucosyl unit. Peaks 8 and 10 were identified respectively as luteolin and kaempferol glucosides both showing pseudomolecular ion [M − H]^−^ at *m/z* 447 (MS^2^ unit at *m/z* 285). Peak 6 was identified as diosmin, characterized by the typical mass spectra in negative mode with the pseudomolecular ion [M − H]^−^ at *m/z* 607. Peaks 7, 9, 11, 12, 13, 14, and 15 were identified as naringin, myricetin, naringenin, quercetin, luteolin, kaempferol, and apigenin, presenting pseudomolecular ions [M − H]^−^ at *m/z* 579, 317, 271, 301, 285, 285, and 269, respectively.

The qualitative and quantitative profile of identified compounds (Table 2) varied between seeds, hypocotyls, cotyledons, and leaves of the three *C. cardunculus* analyzed genotypes. Vitexin (apigenin-8-*C*-glucoside) concentration in tissues and genotypes showed an increase during plant development in the three genotypes, reaching a maximum in leaves with the highest values in “Bianco Avorio” (2107.906 µg g^−1^) (dw), followed by “Spagnolo” (2100.344 µg g^−1^) (dw) (Table 2 and Supplementary Table S3). Similar behavior was also observed for luteolin 7-*O*-glucoside, quercetin 3-glucoside, luteolin, and kaempferol (Supplementary Table S3), possibly suggesting a developmental regulation of their production. In all tissues and genotypes, chlorogenic acid was the most represented compound, reaching concentrations between 786.031 and 3735.790 µg g^−1^ (dw). Leaves accumulated high levels of chlorogenic acid in all genotypes, especially in “Spagnolo”, though the highest concentration for “Gigante” and “Bianco Avorio” was detected in hypocotyls (Supplementary Table S3). Phenylpropanoids represent essential constitutive and inducible defenses and are crucial for the safe growth and development of the plant. Therefore, these metabolites are usually allocated to protect tissues more exposed to biotic and abiotic stressors [50]. Consistently, the accumulation of total phenols was found to be high in leaves. The high accumulation of CQA precursors and other phenylpropanoids detected in hypocotyls and, to a lower extent, in cotyledons may be associated with the physiological function of these tissues. In particular, hypocotyls could be actively involved in metabolites transport to new growing tissues, actively translocating phenolics to new leaves, whereas the cotyledons could go through metabolic events to mobilize storage reserves [51]. In our tissues and genotypes, we did not detect di-caffeoyl quinic acids. Therefore, in our conditions di-caffeoyl quinic acid isomers, if any, were below the detection limit. We hypothesize that the accumulation of these compounds might depend on the physiological state of the plant and be developmentally regulated, as already reported [12]. These interesting findings remain to be further elucidated and will be the subject of future investigations.

### 3.2. Qualitative and Quantitative Profile of (Seeds, Hypocotyls, Cotyledons, and Leaves) Oil 

**Figure 1:**
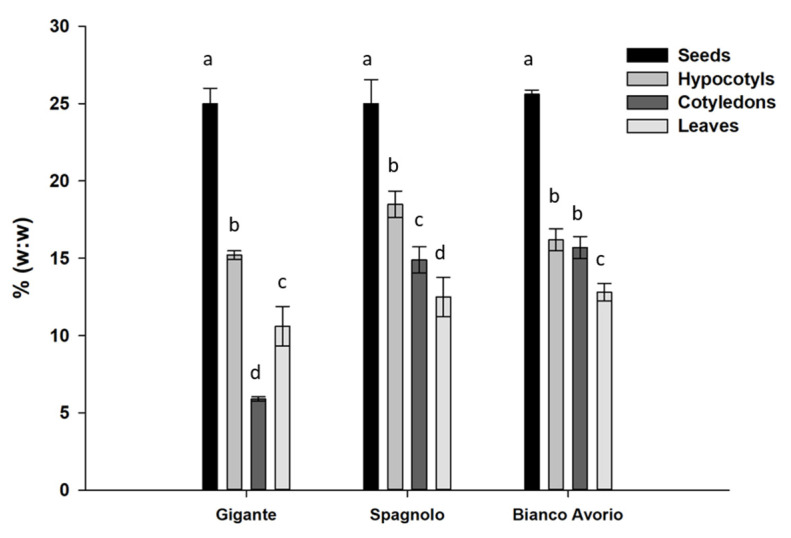
Percent oil content (*w:w*) in the n-exan extract in cotyledons, leaves, hypocotyls, and seeds of cardoon genotypes. Each value represents the mean of three biological and two technical replicates. Different letters denote significant differences among tissues by analysis of variance [ANOVA]. Statistical significance was defined as *p* < 0.05, using Tukey’s post hoc test for mean separation.

Regarding the oil content, the results showed that the highest oil concentration is reached in the seeds, with an average level of 25.2%, in agreement with previous reports [52] but without substantial differences between the three analyzed genotypes (Figure 1). The lowest average level was found in the leaves for all genotypes, with the exception of “Gigante”, for which the lowest average oil content was detected in cotyledons.

The fatty acid profile of the oil represents the main factor that determines the choice of using the oil either for nutritional or industrial purposes. [53] found that cardoon oil is a rich source of unsaturated fatty acids such as linoleic and oleic acids (44.5% and 42.6%, respectively), whereas saturated fatty acids such as palmitic and stearic acid were detected in lower amounts (9.8% and 3.1%, respectively). Since the oil composition is deeply influenced by genotype, climatic factors, and geographical origin [54], growing the three cardoon genotypes in the same controlled greenhouse conditions should have allowed to highlight only differences due to genetic variability. Our results are in agreement with those reported by [53,55,56], highlighting a considerable variation in fatty acid profile between the analyzed genotypes, as shown in Table 3.

Palmitic acid was the most representative fatty acid in all the analyzed tissues and genotypes (Table 3 and Supplementary Table S4). In contrast to what was reported in the literature [57], in all the studied tissues and genotypes we did not detect stearic acid. Oleic acid content was found to vary from 6.54% to 19.27% and reached the highest amount in the oil extracted from the seeds of the genotype “Gigante”, but also in the other genotypes its content was higher in seeds than in all the other analyzed tissues (Table 3 and Supplementary Table S4). The content of linoleic acid varied from 9.20% to 45.43%, with hypocotyls of “Bianco Avorio” showing the highest concentration. Although the hypocotyls of the three genotypes accumulated the highest content of linoleic acid, it is worth noting that a considerable amount of linoleic acid was detected also in “Spagnolo” leaves (Table 3 and Supplementary Table S4). Finally, the content of palmitic acid varied from 34.33 to 63.32% and the highest amount was observed for the oil extracted from the cotyledons of the genotype “Gigante”. The fatty acids profile differences had a significant impact on the ratio of polyunsaturated/saturated fatty acids, ranging from 0.38 to 1.42, and highlighted the highest ratio for the oil extracted from hypocotyls of all genotypes analyzed. Surprisingly, the same was found for the oil extracted from the leaves of the genotype “Spagnolo”. This value is an important parameter in determining the nutritional value and functional properties of food products and oils as oils with a polyunsaturated/saturated fatty acids ratio greater than one are considered valuable edible oils.

### 3.3. Transcriptional Analysis of Key Biosynthetic Genes in Chlorogenic Acid and Monounsaturated Fatty Acids

**Figure 2:**
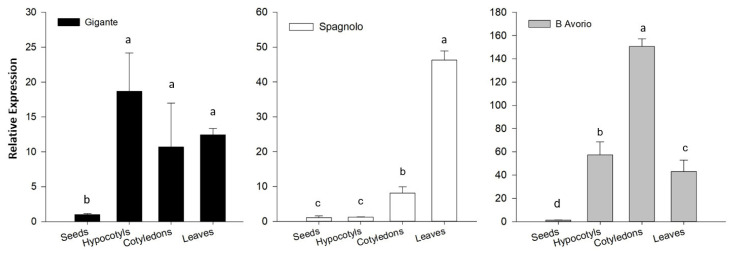
Transcriptional analysis of the Cc*HQT* gene by qRT-PCR in seeds, hypocotyls, cotyledons and leaves of “Gigante”, “Spagnolo” and “Bianco Avorio” cultivated cardoon genotypes. Results are expressed as fold changes relatively to seeds, used as internal calibrator of gene expression for each genotype. Each value represents the mean ± SD of three biological and two technical replicates. Different letters denote a significant difference between tissues by analysis of variance [ANOVA]. Statistical significance was defined as *p* < 0.05, using the Tukey’s post hoc test for mean separation.

**Figure 3:**
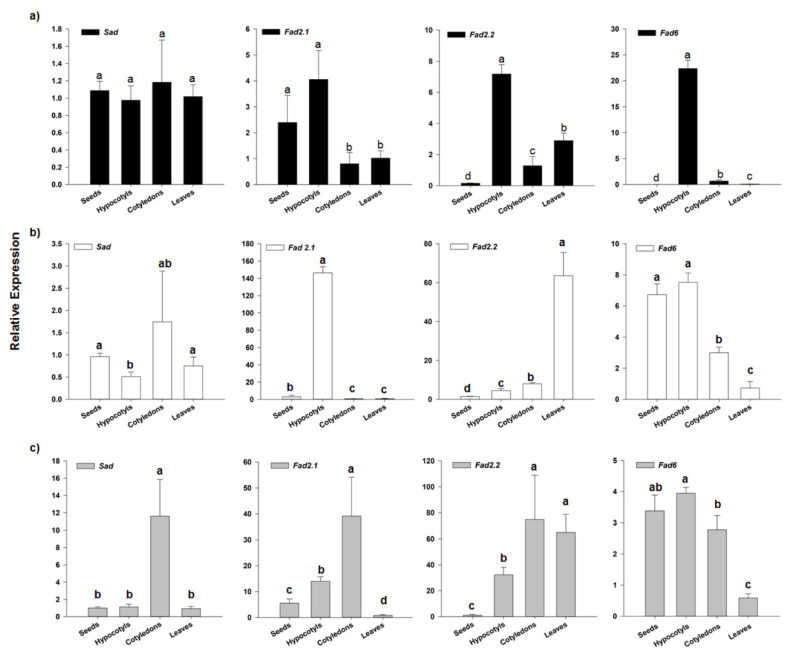
Transcriptional profiling of *SAD*, *FAD2.1*, *FAD2*.2 and *FAD6* genes by qRT-PCR in seeds, hypocotyls, cotyledons and leaves of cardoon genotypes. (**a**) “Gigante”, (**b**) “Spagnolo”, (**c**) “Bianco Avorio”. Results are expressed as fold changes relatively to the tissue with the lowest expression level, used as internal calibrator for each gene within each genotype. Each value represents the mean ± SD of three biological and two technical replicates. For each gene, different letters denote a significant difference between tissues within each genotype by analysis of variance [ANOVA]. Statistical significance was defined as *p* < 0.05, using the Tukey’s post hoc test for mean separation.

To gain insight into the metabolic activities of cardoon tissues and organs, we analyzed transcripts abundance for *HQT* (hydroxycinnamoyl quinate transferase; Figure 2), as a key gene in the biosynthesis of chlorogenic acid, and for *SAD* (stearoyl acid desaturase) and *FAD2* (fatty acid desaturase) genes (Figure 3), involved in oleic and linoleic acid formation, respectively. We identified the genes coding for enzymes involved in the biosynthesis of CGAs and mono-unsaturated fatty acids from deposited sequences. *HQT* was previously isolated and characterized in *C. cardunculus*. var *altilis* and globe artichoke [23,24], whereas for putative *SAD* and *FAD2* genes, sequences were retrieved from BLAST searches in the *C. cardunculus* database (PRJNA453787) and chosen based on the high nucleotidic and proteic identity to orthologous genes in closely related Asteraceae and other oleaginous species. Regarding *SAD*, we found only two cardoon sequences, highly similar to each other, and coding for proteins closely related to characterized *Lactuca sativa* (XM 023873428.1) and *Olea europaea* var. *sylvestris* (XM 023023317.1) *SAD2*. As regards *FAD* genes, two main oleate desaturase genes occur in oleaginous plants, namely the microsomal and plastidial types [32]. In detail, two microsomial genes are characterized by tissue specificity, the seed type *FAD2.1* and the house-keeping *FAD2.2*, along with the plastidial one, known as *FAD6*. We selected three *FAD2* representatives: *CcFAD2.1* (70.08% to GmFAD2-l, AAB00859, and 77.89% to *Ha*FAD2-1, AAL68981.1), *CcFAD2.2* (90.00% to *Cp*FAD2-2, CAA76157 and 90.82% to *Ha*Fad2.2, AAL68982.1), and *FAD6* (92.88% to *Aa*FAD6, PWA37615 and 83% to *Tc*FAD6, GEU42198). Although gene expression analyses were carried out for only one gene within the phenylpropanoid pathway and for *SAD* and three *FAD2* isoforms for fatty acids biosynthesis, the selected genes represent key steps in the respective pathways. Gene transcripts were detected for all the tissues analyzed, and transcript abundances varied in tissues and genotypes underlining a tight regulation for the accumulation of these metabolites. 

#### 3.3.1. *HQT* Expression Analysis

In details, transcriptional analysis of *HQT* gene expression (Figure 2) was consistent with CQA accumulation among the genotypes (Table 2), which was higher in “Bianco Avorio” than “Gigante” and “Spagnolo”. Nevertheless, within each genotype, *HQT* expression did not reflect CQA accumulation for most of the analyzed tissues, in particular seeds and cotyledons. In all genotypes, *HQT* expression was barely detectable in seeds and much higher in cotyledons, while CGA levels were comparable to other tissues in seeds and the lowest in cotyledons (Figure 2 and Table 2). Due to their quiescent state, mature seeds might have a reduced transcriptional activity not temporally related with CGA accumulation, whose biosynthesis is highly regulated during seed development in order to guarantee a safe storage of energy in form of chlorogenic acid at seed maturity. Instead, in cotyledons, the high *HQT* transcriptional activity might be needed to provide high levels of CGA, which is degraded to provide lignin-precursors necessary for cotyledonary cell wall formation [58]. Regarding *HQT* expression in the other tissues we cannot exclude that additional pathways can contribute to CGA synthesis, as reported in other species [59]. Moreover, expression analysis can be only indicative of the transcriptional dynamics linked to CQA accumulation, which is mostly dependent on the enzymatic activity, as reported in other species [59,60]. We believe that *HQT* expression is also the result of a fine regulatory scheme, since this key gene is rate limiting and we cannot exclude that a feedback regulation is involved in its transcriptional rate, as recently reported [61].

#### 3.3.2. *SAD* and *FAD2* Expression Analysis

Transcriptional analysis of key biosynthetic genes in oleic and linoleic acid formation revealed that *SAD* expression was low in seeds of all three genotypes. In “Gigante” and “Spagnolo” it was low also in the other analyzed tissues, whereas in “Bianco Avorio” *SAD* transcription was remarkable in cotyledons but not consistent with quantitative oleic acid detection (Figure 3). The weak correlation between oleic acid accumulation and *SAD* expression might be related to the developmental seed stage investigated in this study. In mature seed, oleic acid might have reached an accumulation threshold, driving a feedback inhibition of *SAD* expression in favor of oleic to linoleic conversion. This hypothesis is consistent with the higher content of linoleic acid in almost all the analyzed tissues and genotypes. Regarding the expression analysis of the FAD isogenes, we found that the expression levels of the three *FAD* genes were higher in hypocotyls than in the other tissues in the “Gigante” genotype. Consistently, high linoleic acid accumulation was detected in hypocotyls of this genotype (Table 3), possibly resulting from the contribution of all the *FAD* isoforms. In “Spagnolo”, *FAD2.1* and *FAD2*.2 showed a clear tissue-specificity, being highly expressed in hypocotyls and leaves, respectively, and we might hypothesize that each isoform contributed to the higher linoleic acid content of these two tissues in this genotype. As for *FAD6*, transcripts accumulation was higher in “Spagnolo” seeds and hypocotyls than in cotyledons and leaves. Regarding “Bianco Avorio”, high *FAD2.1* expression was detected in cotyledons, where also *FAD2*.2 accumulated at high levels, similarly to leaves, whereas *FAD6* was higher in seeds and hypocotyls. The distribution of *FAD* transcripts, in particular for *FAD2*.2, was in accordance with published literature in other oleaginous plants, indicating it as constitutive gene [59]. Indeed, *FAD2*.2 was expressed in all tissues, except for dormant seeds, and, according to transcriptional data, could play a main role in linoleic acid formation in “Spagnolo” and “Bianco Avorio”. Nevertheless, functional studies are necessary to ascertain the role of each gene in this species and whether it might be affected by genotype differences. Overall, our results reflect the harmonic interplay between *SAD* and *FAD* genes, the lower level of *SAD* being seemingly compensated by the higher expression of *FAD* genes, which finally regulates the MUFA/ PUFA ratio in all the cardoon tissues.

### 3.4. Antioxidant Activity of Polyphenolic Extracts

The results obtained for antioxidant activity of cultivated cardoon samples by the ABTS, FRAP and DPPH assays are presented in Table 4 and expressed as TEAC (mmol kg^-1^ dw). The measurement of the antioxidant activity of food is carried out using several methods since it is influenced by various factors. The most commonly used for the evaluation of the antioxidant potential are DPPH radical scavenging, ABTS decolorization and FRAP assays due to their simplicity, stability, accuracy and reproducibility [62].

As for seeds, FRAP values ranged from 49.83 ± 1.28 to 107.73 ± 1.30 mmol kg^−1^ dw, whereas ABTS values ranged from 87.24 ± 53.31 to 148.04 ± 1.13 mmol kg^-1^ dw and DPPH values ranged from 49.49 ± 0.27 to 143.87 ± 0.94 mmol kg^-1^ dw. Antioxidant activity showed significant differences between the tested genotypes, with “Bianco Avorio” having the overall highest antioxidant activity. Our results are in accordance with the results obtained by [11], reporting significant differences between wild and cultivated cardoon genotypes, as well as between hydromethanolic extracts and its residues. [63], similarly to our data, estimated that seed extracts of wild cardoons showed a high DPPH^•^ scavenging activity and a considerable ABTS^•+^ radical scavenging capacity. Moreover, the correlation coefficient between phenolic contents and TEAC was highly significant especially in the case of ABTS and FRAP (*r*^2^ = 0.82 and 0.94, respectively), indicating that polyphenolics may play an important role in free radical cations scavenging and ferric reducing antioxidant power.

Similar results were obtained for hypocotyls, where the correlation coefficients between phenolic contents and TEAC values were highly significant (*r*^2^ = 1, 0.94, and 0.95, respectively for DPPH, ABTS and FRAP). The results obtained showed a remarkable antioxidant activity of the genotype “Bianco Avorio” also for the hypocotyls and the highest antioxidant activity of polyphenols especially as free radical cation scavengers, being the antioxidant activity evaluated with the ABTS assay the highest. For cardoon hypocotyls, in fact, FRAP values ranged from 57.94 ± 1.043 to 79.76.73 ± 2.69 mmol kg^−1^ dw, ABTS values ranged from 317.21 ± 2.071 to 667.37 ± 1. mmol kg^−1^ dw and finally DPPH values ranged from 37.54 ± 0.311 to 60.87 ± 0.106 mmol kg^-1^ dw. These results highlight a significant variability in nutraceutical potential between genotypes.

For cotyledons, the results showed a significant variability in antioxidant potential between genotypes (Table 4). FRAP values ranged from 47.78 ± 0.987 to 79.148 ± 2.876 mmol kg^−1^ dw, whereas ABTS values ranged from 412.113 ± 0.112 to 543.012 ± 0.765 mmol kg^-1^ dw and finally DPPH values ranged from 19.192 ± 0.123 to 63.951 ± 0.543 mmol kg^−1^ dw. The highest antioxidant capacities were recorded for the “Spagnolo” genotype using the DPPH and FRAP methods while, in analogy with the results described so far, “Bianco Avorio” showed the highest scavenger capacity against the ABTS radical cations. In literature, the deposition of phenolic polymers was reported for Coffea arabica cotyledons during development, suggesting that lignification may occur via the utilization of the stored 5-O-caffeoylquinic acid. This result would explain the lower antioxidant activity of the cotyledons also associated with a low level of chlorogenic acid. Finally, for cardoon leaves, FRAP values ranged from 62.45 ± 0.432 to 91.675 ± 0.761 mmol kg^−1^ dw, whereas ABTS values ranged from 510.315 ± 0.766 to 771.412 ± 0.665 mmol kg^−1^ dw and finally DPPH values ranged from 27.167 ± 0.551 to 91.856 ± 0.116 mmol kg^−1^ dw.

The nutraceutical properties of leaves of artichoke and cardoon are linked to their specific chemical composition. Caffeoylquinic acids, followed by flavonoids, are the main phenolic compounds in leaves, among which chlorogenic acid is the most abundant. The same order of activity was found in the DPPH and FRAP assays, but the absolute values were lower than those found in the ABTS assay. According to literature data [64] ABTS assay is the most sensitive assay, as the working solution is soluble in aqueous and organic solvents, at several pH values, and the quenching reaction of free radicals is faster than other methods. The antioxidant capacity of cardoon leaf extracts was reported [12] and different artichoke genotypes were studied in terms of their antioxidant capacity [65]. Antioxidant activity measured on the leaf polyphenolic extracts was remarkably higher than that reported in literature, on the other hand little change in experimental conditions can widely influence the results making the comparison only qualitative. For leaves, the correlation coefficients between phenolic contents and TEAC values were mildly significant (*r*^2^ = 0.98, 0.83 and 0.70, respectively for DPPH, ABTS and FRAP), with the genotype “Bianco Avorio” showing the highest antioxidant activity especially as ABTS radical scavenger.

## 4. Conclusions

In conclusion, the results presented in this work provide a detailed analysis of tissues composition of cardoon, enabling to elucidate value-added products accumulation and distribution during plant development and hence contributing to optimize the sustainable use of this natural resource. Moreover, transcriptional and metabolic screening could be useful for a careful selection of the genotype.

## Figures and Tables

**Table 1:** Retention time and exact mass spectra data of cultivated cardoon polyphenols investigated by UHPLC-HRMS Orbitrap.

Peak Number	Polyphenol	Retention Time (min)	Chemical Formula	Theoretical Mass (*m/z*)	Measured Mass (*m/z*)	Accuracy (ppm)
13	Luteolin	10.99	C_15_H_10_O_6_	285.04046	285.04071	0.88
15	Apigenin	11.45	C_15_H_10_O_5_	269.04555	269.04605	1.86
6	Diosmin	9.96	C_28_H_32_O_15_	607.16684	607.16821	2.26
5	Apigenin-8-*C*-glucoside	9.89	C_21_H_20_O_10_	431.09837	431.09973	3.15
11	(+/−) Naringenin	10.58	C_15_H_12_O_5_	271.06120	271.06165	1.66
4	Quercitin-3-*O*-glucoside	9.71	C_21_H_19_O_12_	463.08820	463.08926	2.29
12	Quercetin	10.71	C_15_H_10_O_7_	301.03538	301.03540	0.07
14	Kaempferol	11.31	C_15_H_10_O_6_	285.04046	285.04077	1.09
9	Myricetin	10.06	C_15_H_10_O_8_	317.03029	317.03119	2.84
7	Naringin	10.01	C_27_H_32_O_14_	579.54123	579.54162	0.67
10	Kaempferol-3-*O*-glucoside	10.11	C_21_H_20_O_11_	447.09328	447.09396	1.52
8	Luteolin-7-*O*-glucoside	10.05	C_21_H_20_O_12_	447.09328	447.09311	−0.38
3	*p*-Coumaric acid	8.66	C_9_H_8_O_3_	163.04007	163.04056	3.00
2	Chlorogenic acid	7.85	C_16_H_18_O_9_	353.08781	353.08856	2.12
1	4-Hydroxybenzoic acid	6.76	C_7_H_6_O_3_	137.02442	137.02489	3.43

**Table 2:** Polyphenols content in seeds, hypocotyls, cotyledons, and leaves of “Gigante”, “Spagnolo” and “Bianco Avorio” genotypes detected by HRMS-Orbitrap. Values are expressed in mg g^−1^ (dw). Each value represents the mean of three biological and two technical replicates. Different letters denote a significant difference between genotypes within each tissue by analysis of variance [ANOVA]. Statistical significance was defined as *p* < 0.05, using Tukey’s post hoc test for mean separation. nd: not detected.

Phenolic Compounds	SEEDS			HYPOCOTYLS			COTYLEDONS			LEAVES		
	Gigante	Spagnolo	B. Avorio	Gigante	Spagnolo	B. Avorio	Gigante	Spagnolo	B. Avorio	Gigante	Spagnolo	B. Avorio
4-hydroxy benzoic acid	2.750a	2.510b	2.040c	5.620a	3.760c	3.910b	12.592b	8.795c	16.771a	1.221a	0.661b	0.329c
Vitexin	107.300a	91.400b	83.700c	121.700b	111.710c	250.110a	350.432c	876.529b	1182.892a	528.396c	2100.344b	2107.906a
luteolin-7-*O*-glucoside	1.470b	2.760a	0.910c	1.610c	8.610b	12.700a	18.092c	34.299a	24.086b	81.922c	122.687a	88.773b
naringin	10.900c	11.700b	14.400a	5.710c	51.700b	67.100a	14.599b	438.433a	436.022a	0.251b	0.357a	0.038c
chlorogenic acid	1036.310c	1201.990b	1430.460a	3461.130b	1006.780c	3735.790a	786.031a	769.301a	463.390b	1468.968b	2467.679a	2637.733a
coumaric acid	1.790a	1.840a	1.820a	3.620a	3.410a	3.720a	0.180a	0.080b	0.080b	0.080c	0.560a	0.200b
quercetin-3-glucoside	0.090c	0.270b	1.160a	0.700b	0.180c	1.180a	1.587b	2.670a	1.970b	3.386a	3.618a	2.050b
diosmin	0.050a	nd	nd	nd	nd	nd	nd	nd	nd	nd	nd	nd
kaempferol-3-*O*-glucoside	2.180a	2.140a	2.200a	4.910ab	4.460b	5.000a	0.420b	0.780a	0.860a	9.960a	9.960a	6.720b
myricetin	0.440a	0.440a	0.450a	0.880a	0.890a	0.880a	2.470c	3.110b	8.470a	6.884a	6.063b	5.493c
naringenin	1.730a	1.610b	1.680ab	3.130a	3.110a	3.110a	0.120a	nd	0.040b	0.160c	0.320b	0.500a
luteolin	0.320a	0.020b	0.020b	0.040b	0.200a	nd	11.700c	37.137a	31.464b	56.040c	84.640a	72.671b
kaempferol	0.340a	0.020b	0.020b	0.040b	0.240a	nd	3.600c	4.300b	7.900a	4.800c	7.360a	5.780b
quercetin	0.820	nd	nd	nd	1.640	nd	nd	0.020a	0.020a	36.580a	21.300b	14.160c
apigenin	0.240a	0.040b	0.020c	nd	0.080a	0.040b	nd	4.580b	7.040a	2.260c	3.710b	8.080a
Total polyphenols	1166.730c	1316.740b	1538.880a	3609.090b	1196.770c	4083.540a	1201.823b	2180.034a	2181.005a	2200.908b	4829.259a	4950.433a

**Table 3:** GC/MS analysis of fatty acids composition in cardoon genotypes (expressed as % of the total fatty acid composition). Each value represents the mean of three biological and two technical replicates. Different letters denote a significant difference between genotypes within each tissue by analysis of variance [ANOVA]. Statistical significance was defined as *p* < 0.05, using Tukey’s post hoc test for mean separation. n.d.: not detected.

Fatty Acids	Seeds			Hypocotyls			Cotyledons			Leaves		
	Gigante	Spagnolo	B. Avorio	Gigante	Spagnolo	B. Avorio	Gigante	Spagnolo	B. Avorio	Gigante	Spagnolo	B. Avorio
Pentadeconoic (C15:0)	0.123c	0.283a	0.173b	0.172b	0.182a	0.143c	1.852a	0.578c	0.711b	0.985a	0.441c	0.611b
Palmitic (C16:0)	59.868a	44.265b	56.708a	49.361a	47.722a	40.791b	63.320a	44.594c	50.348b	44.484b	34.330c	61.643a
Margaric (C17:0)	0.694b	1.25a	0.797b	0.109a	0.117a	0.098b	2.253a	0.639b	0.642b	0.882a	0.571b	0.608b
Nonadecanoic (C19:0)	0.013b	0.024a	0.01b4	n.d.	n.d.	n.d.	n.d.	0.252	n.d.	0.289	n.d.	0.187
Arachidic (C20:0)	4.446ab	4.427a	3.4b	n.d.	n.d.	0.659	n.d.	8.176a	5.157b	7.635a	3.852c	5.309b
Behenic (C22:0)	0.634a	0.635a	0.489b	0.271b	0.431a	0.315b	1.976b	3.767a	1.459c	4.162a	2.103c	3.463b
Lignoceric (C24:0)	0.457a	0.246b	0.432a	0.326	n.d.	n.d.	2.508b	5.408a	0.979c	5.219b	n.d.	5.706a
Cerotic (C26:0)	0.052	n.d.	n.d.	n.d.	n.d.	n.d.	0.465a	4.414a	n.d.	3.977	n.d.	n.d.
Melissic (C30:0)	0.015	0.03	n.d.	n.d.	n.d.	0.282	n.d.	n.d.	0.557	n.d.	n.d.	n.d.
Palmitoleic (C16:1)	0.372b	0.816a	0.348b	n.d.	0.135	n.d.	0.607a	n.d.	0.439b	1.452a	0.420c	0.516b
Hexadicadienoic (C16:2)	0.006b	1.389a	0.009b	n.d.	n.d.	n.d.	n.d.	n.d.	n.d.	n.d.	0.088	n.d.
Hexadicatrienoic (C16:3)	n.d.	n.d.	n.d.	n.d.	n.d.	n.d.	9.774	n.d.	n.d.	n.d.	n.d.	n.d.
Oleic (C18:1)	19.271a	18.395b	15.828c	6.54c	7.869b	12.278a	n.d.	9.737a	8.240b	10.524b	15.519a	6.592c
Linoleic (C18:2)	13.684c	27.34a	21.212b	37.892b	38.954b	45.434a	8.935c	12.997b	24.714a	12.806b	37.460a	9.197c
Linolenic (C18:3)	0.355a	0.441a	0.021b	5.259a	4.588b	n.d.	8.312ab	9.438a	6.644b	7.584a	5.215c	6.169b
Nonadecenoic (C19:1)	0.01	0.018	n.d.	n.d.	n.d.	n.d.	n.d.	n.d.	n.d.	n.d.	n.d.	n.d.
Gadoleic (C20:1)	n.d.	0.441b	0.566a	0.07	n.d.	n.d.	n.d.	n.d.	0.110	n.d.	n.d.	n.d.

**Table 4:** Antioxidant activity in seeds, hypocotyls, cotyledons and leaves of three cultivated cardoon genotypes. Each value represents the mean ± SD of three biological and two technical replicates. Different letters denote a significant difference between genotypes within each tissue by analysis of variance [ANOVA] Statistical significance was defined as *p* < 0.05, using the Tukey’s post hoc test for mean separation.

GENOTYPE		DPPH	ABTS	FRAP
		mmol trolox kg^−1^	mmol trolox kg^−1^	mmol trolox kg^−1^
Seeds	GIGANTE	49.492 ± 0.275c	87.237 ± 3.312c	49.833 ± 1.283c
	SPAGNOLO	143.871 ± 0.942a	135.413 ± 1.119b	59.848 ± 3.340b
	BIANCO AVORIO	137.946 ± 0.265b	148.037 ± 1.127a	107.728 ± 1.303a
Hypocotyls	GIGANTE	57.149 ± 0.377b	667.365 ± 1.036a	72.000 ± 0.954b
	SPAGNOLO	37.543 ± 0.311c	317.206 ± 2.071c	57.939 ± 1.043c
	BIANCO AVORIO	60.866 ± 0.106a	631.482 ± 8.285b	79.758 ± 2.694a
Cotyledons	GIGANTE	19.192 ± 0.123c	487.277 ± 2.111b	47.776 ± 0.987c
	SPAGNOLO	63.951 ± 0.543a	412.113 ± 0.112c	79.148 ± 2.876a
	BIANCO AVORIO	55.126 ± 0.156b	543.012 ± 0.765a	57.122 ± 1.832b
Leaves	GIGANTE	27.167 ± 0.551c	510.315 ± 0.765c	62.450 ± 0.432c
	SPAGNOLO	81.543 ± 0.111b	654.216 ± 0.981b	97.659 ± 0.876a
	BIANCO AVORIO	91.856 ± 0.116a	771.412 ± 0.665a	91.675 ± 0.761b

## Supplementary Materials

The following are available online at https://www.mdpi.com/2076-3921/9/11/1096/s1.
