# Supplementary material for: Changes in Phenolics and Fatty Acids Composition and Related Gene Expression during the Development from Seed to Leaves of Three Cultivated Cardoon Genotypes
Source: Antioxidants (Basel). 2020 Nov 8;9(11):1096. doi: 10.3390/antiox9111096 (PMC7695130; doi:10.3390/antiox9111096)
Supplement: Supplementary file 1 [file antioxidants-09-01096-s001.pdf]

Changes in phenolics and fatty acids composition and related gene expression during the development from seed to leaves of three cultivated cardoon genotypes

Giulia Graziani[1,*,†], Teresa Docimo[2,†], Monica De Palma[2], Francesca Sparvoli[3], Luana Izzo[1], Marina Tucci[2,*], Alberto Ritieni[1,4]
[1] Department of Pharmacy, University of Naples Federico II, Via Domenico Montesano 49, 80131 Naples, Italy; giulia.graziani@unina.it; luana.izzo@unina.it; alberto.ritieni@unina.it
[2] Institute of Bioscience and Bioresources, Consiglio Nazionale delle Ricerche, via Università 133, 80055 Portici, Italy; teresa.docimo@ibbr.cnr.it; monica.depalma@ibbr.cnr.it; mtucci@unina.it
[3] Institute of Agricultural Biology and Biotechnology, Consiglio Nazionale delle Ricerche, Via E. Bassini 15, 20133 Milan, Italy; sparvoli@ibba.cnr.it
[4] Unesco Chair for Health Education and Sustainable Development, 80131 Naples, Italy
* Correspondence: giulia.graziani@unina.it; mtucci@unina.it
† These authors contributed equally to this work

Supplementary Information

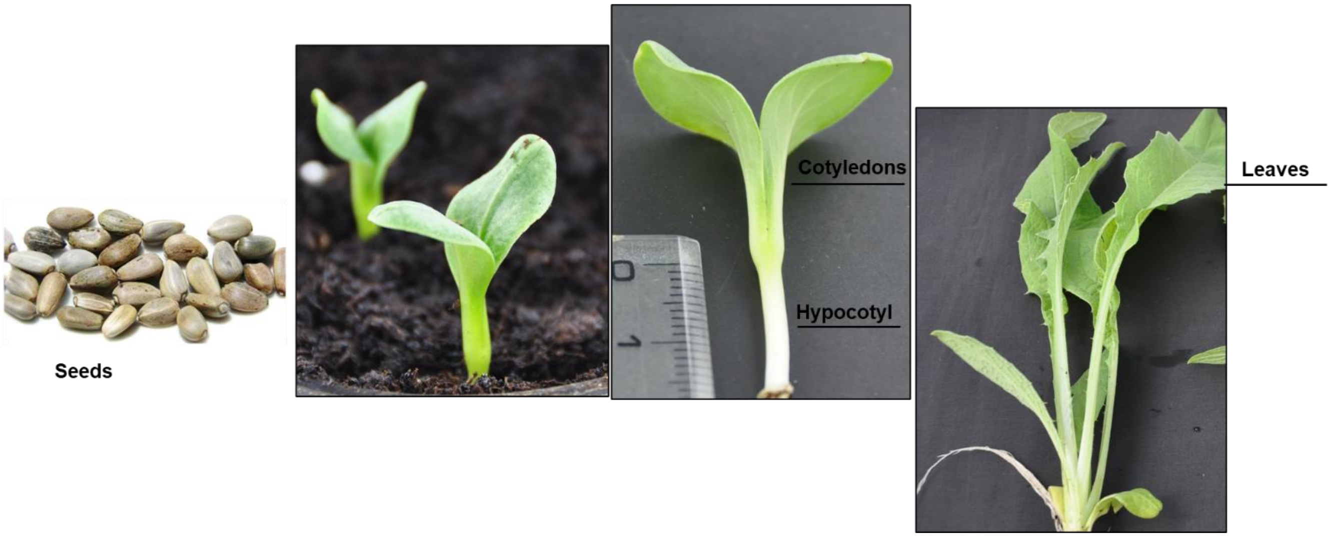

Supplementary Figure 1. Tissues of *Cynara cardunculus* var *altilis* analyzed in this study

Supplementary Table 1. Primers used in this study for qRT-PCR analysis

Gene and accession number	Primer Sequencee (5'-3')	Amplicon (bp)	Tm (C°)
CcAct	Fw- TAC TTT CTA CAA CGA GCT TC	107	60
(XM_025103545)	Rev- ACA TGA TTT GAG TCA TCT TC		60
CcHQT	Fw- TCA CAC AGG TTA CAC GCT TCA ACT G	110	63
(DQ915589.1)	Rev- GGG CTT TAT CGG ACC ATG TAT TGA T		61.3
CcFAD2.1	Fw- TCT TTC AAC GTG TCT GGA AGA CCC	110	60
(XM_025119818)	Rev- CCC ACG TCA GAA AGC CAA ATT TG		59
CcFAD2.2	Fw- AAT CTT CAT CTC CGA CGC CG	105	59.4
(XM_025117942.1)	Rev- GTT CAC CAC AAG CAA CGG TC		59.4
CcFAD6	Fw- GGA CGC AAT CAA GCC AGT TT	108	57.3
(XM_025111323)	Rev- CGC TAT CTT CAT CGG GCT CA		59.4
SAD			
(XM_025110267) and	Fw- GTG ACC ACC AAT CAC CAC CA	110	59.4
(XM_025110975.1)	Rev- GGA AAG GGA GGA TGG ATT GT		57.3

Supplementary Table 2 Linearity, LOD, LOQ, precision and recovery for the 15 authentic standards (n = 5)

Polyphenol	LOD	LOQ	linearity (R^2)	recovery % (n=3)			Intra-day precision (RSD,%; (n=3)	Intra-day precision (RSD,%; (n=3)
	ng/g			1 mg/kg	10 mg/kg	50 mg/kg	1 mg/kg	
Luteolin	0.04	0.12	0.991	91.3	99.5	105.31	5	6
Apigenin	0.05	0.14	0.992	99.34	91.56	102.34	3	8
Diosmin	0.05	0.16	0.995	99.12	98.13	102.45	6	9
Apigenin-8-C-glucoside	0.04	0.12	0.995	100.12	100,0	102.32	7	10
(+/-) Naringenin	0.04	0.12	0.991	96.34	98.38	103.56	5	5
Quercitin-3-O-glucoside	0.05	0.14	0.994	98.45	99.47	103.56	4	7
Quercetin	0.04	0.13	0.994	99.12	100.56	102.23	2	8
Kaempferol	0.04	0.12	0.995	100.23	99.45	104.56	7	9
Myricetin	0.03	0.1	0.991	100.58	99.91	104.49	5	7
Naringin	0.05	0.14	0.997	98.45	99.19	105.91	6	6
Kaempferol-3-O-glucoside	0.03	0.1	0.987	92.98	95.01	100.12	3	8
Luteolin-7-O-glucoside	0.05	0.14	0.992	99.91	100.12	102.51	5	9
p-Coumaric acid	0.05	0.14	0.989	99.78	100.19	105.51	5	6
Chlorogenic acid	0.04	0.13	0.992	100.29	102.61	103.12	8	9
4-Hydroxybenzoic acid	0.04	0.12	0.991	102.87	99019,0	101.81	6	9

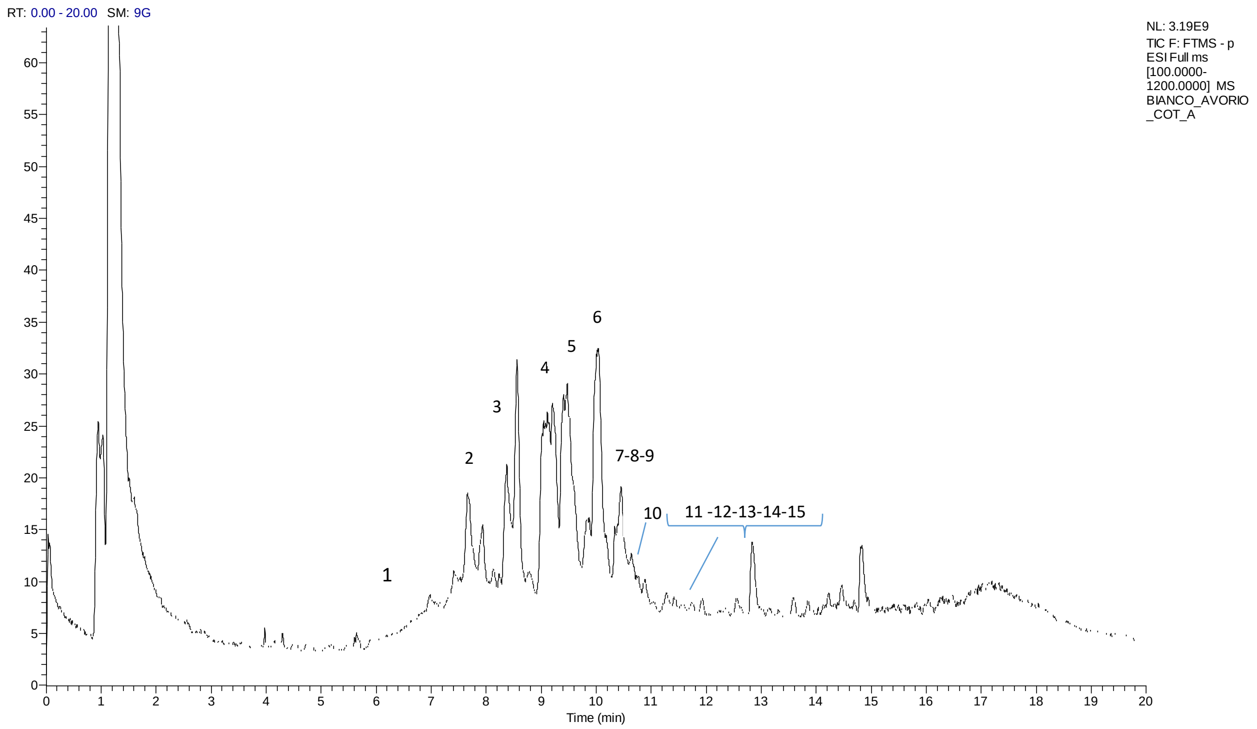

Supplementary Figure 2. A typical full-scan MS chromatogram of a cardoon sample (cotyledons). Peak assignments are reported below.

1	4-Hydroxybenzoic acid
2	Chlorogenic acid
3	p-Coumaric acid
4	Quercitin-3-O-glucoside
5	Apigenin-8-C-glucoside
6	Diosmin
7	Naringin
8	Luteolin-7-O-glucoside
9	Myricetin
10	Kaempferol-3-O-glucoside
11	(+/-) Naringenin
12	Quercetin
13	Luteolin
14	Kaempferol

Supplementary Table 3. Polyphenols content in seeds, hypocotyls, cotyledons and leaves of "Gigante", "Spagnolo" and "Bianco Avorio" cultivated cardoon genotypes detected by HRMS-Orbitrap. Values are expressed in mg g^{-1} (dw). Each value represents the mean of three biological and two technical replicates. Different letters denote a significant difference between tissues of each genotype by analysis of variance [ANOVA]. Statistical significance was defined as $p < 0.05$, using the Tukey's post hoc test for mean separation.

**Phenolic compounds**	**Gigante**				**Spagnolo**				**B. Avorio**			
	**Seeds**	**Hypocotyls**	**Cotyledons**	**Leaves**	**Seeds**	**Hypocotyls**	**Cotyledons**	**Leaves**	**Seeds**	**Hypocotyls**	**Cotyledons**	**Leaves**
4-hydroxy benzoic acid	2.750c	5.620b	**12.592a**	1.221d	2.510c	3.760b	**8.795a**	0.661d	2.040c	3.910b	**16.771a**	0.329d
Vitexin	107.300d	121.700c	350.432b	**528.396a**	91.400d	111.710c	876.529b	**2100.344a**	83.700d	250.110c	1182.892b	**2107.906a**
luteolin-7-O-glucoside	1.470d	1.610c	18.092b	**81.922a**	2.760d	8.610c	34.299b	**122.687a**	0.910d	12.700c	24.086b	**88.773a**
naringin	**10.900a**	5.710b	**14.599a**	0.251c	11.700c	51.700b	**438.433a**	0.357d	14.400c	67.100b	**436.022a**	0.038d
chlorogenic acid	1036.310c	**3461.130a**	786.031d	1468.968b	1201.990b	1006.780c	769.301d	**2467.679a**	1430.460c	**3735.790a**	463.390d	2637.733b
coumaric acid	1.790b	**3.620a**	0.180c	0.080d	1.840b	**3.410a**	0.080d	0.560c	1.820b	**3.720a**	0.080d	0.200c
quercetin-3-glucoside	0.090d	0.700c	1.587b	**3.386a**	0.270c	0.180d	2.670b	**3.618a**	1.160b	1.180b	**1.970a**	2.050a
diosmin	0.050a	nd	nd	nd	nd	nd	nd	nd	nd	nd	nd	nd
kaempferol-3-O-glucoside	2.180c	4.910b	0.420d	**9.960a**	2.140c	4.460b	0.780d	**9.960a**	2.200c	5.000b	0.860d	**6.720a**
myricetin	0.440d	0.880c	2.470b	**6.884a**	0.440d	0.890c	3.110b	**6.063a**	0.450d	0.880c	**8.470a**	5.493b
naringenin	1.730b	3.130a	0.120c	0.160c	1.610b	3.110a	nd	0.320c	1.680b	**3.110a**	0.040d	0.500c
luteolin	0.320c	0.040d	11.700b	**56.040a**	0.020d	0.200c	37.137b	**84.640a**	0.020c	nd	31.464b	**72.671a**
kaempferol	0.340c	0.040d	3.600b	**4.800a**	0.020d	0.240c	4.300b	**7.360a**	0.020b	nd	**7.900a**	5.780b
quercetin	0.820b	nd	nd	**36.580a**	nd	1.640b	0.020c	**21.300a**	nd	nd	0.020b	**14.160a**
apigenin	0.240b	nd	nd	**2.260a**	0.040d	0.080c	**4.580a**	3.710b	0.020d	0.040c	7.040b	**8.080a**
Total polyphenols	1166.730d	**3609.090a**	1201.823c	2200.908b	1316.740c	1196.770d	2180.034b	**4829.259a**	1538.880d	4083.540b	2181.005c	**4950.433a**

Supplementary Table 4. GC/MS analysis of fatty acids composition in cardoon genotypes (expressed as % of total fatty acid composition). Each value represents the mean of three biological and two technical replicates. Different letters denote a significant difference between tissues of each genotype by analysis of variance [ANOVA]. Statistical significance was defined as $p < 0.05$, using the Tukey's post hoc test for mean separation.

**Fatty acids**	**Gigante**				**Spagnolo**				**Bianco Avorio**			
	**Seeds**	**Hypocotyls**	**Cotyledons**	**Leaves**	**Seeds**	**Hypocotyls**	**Cotyledons**	**Leaves**	**Seeds**	**Hypocotyls**	**Cotyledons**	**Leaves**
Pentadeconoic (C15:0)	0.123d	0.172c	**1.852a**	0.985b	0.283b	0.182c	**0.578a**	**0.441a**	0.173c	0.143d	**0.711a**	0.611b
Palmitic (C16:0)	**59.868a**	49.361b	**63.320a**	44.484b	44.265b	**47.722a**	**44.594ab**	34.330c	**56.708a**	40.791b	50.348b	**61.643a**
Margaric (C17:0)	0.694b	0.109c	**2.253a**	0.882b	**1.25a**	0.117c	0.639b	0.571b	0.797a	0.098d	0.642b	0.608c
Nonadecanoic (C19:0)	0.013b	n.d.	n.d.	**0.289a**	0.024b	n.d.	**0.252a**	n.d.	0.014b	n.d.	n.d.	**0.187a**
Arachidic (C20:0)	4.446b	n.d.	n.d.	**7.635a**	4.427b	n.d.	**8.176a**	3.852c	3.4c	0.659	5.157b	**5.309a**
Behenic (C22:0)	0.634c	0.271d	1.976b	**4.162a**	0.635c	0.431d	**3.767a**	2.103b	0.489c	0.315d	1.459b	**3.463a**
Lignoceric (C24:0)	0.457c	0.326d	2.508b	**5.219a**	0.246b	n.d.	**5.408a**	n.d.	0.432c	n.d.	0.979b	**5.706a**
Cerotic (C26:0)	0.052c	n.d.	0.465b	**3.977a**	n.d.	n.d.	**4.414**	n.d.	n.d.	n.d.	n.d.	n.d.
Melissic (C30:0)	0.015	n.d.	n.d.	n.d.	0.03	n.d.	n.d.	n.d.	n.d.	0.282b	0.557a	n.d.
Palmitoleic (C16:1)	0.372c	n.d.	0.607b	**1.452a**	**0.816a**	0.135c	n.d.	0.420b	0.348c	n.d.	0.439b	**0.516a**
Hexadicadienoic (C16:2)	0.006b	n.d.	n.d.	n.d.	**1.389a**	n.d.	n.d.	0.088b	0.009	n.d.	n.d.	n.d.
Hexadicatrienoic (C16:3)	n.d.	n.d.	9.774	n.d.	n.d.	n.d.	n.d.	n.d.	n.d.	n.d.	n.d.	n.d.
Oleic (C18:1)	**19.271a**	6.54c	n.d.	10.524b	**18.395a**	7.869d	9.737c	15.519b	**15.828a**	12.278b	8.240c	6.592d
Linoleic (C18:2)	13.684b	**37.892a**	8.935c	12.806b	27.34c	**38.954a**	12.997d	37.460b	21.212b	**45.434a**	24.714b	9.197c
Linolenic (C18:3)	0.355c	5.259b	**8.312a**	**7.584a**	0.441d	4.588c	**9.438a**	5.215b	0.021c	n.d.	**6.644a**	6.169b
Nonadecenoic (C19:1)	0.01	n.d.	n.d.	n.d.	0.018	n.d.	n.d.	n.d.	n.d.	n.d.	n.d.	n.d.
Gadoleic (C20:1)	n.d.	0.07	n.d.	n.d.	0.441	n.d.	n.d.	n.d.	**0.566a**	n.d.	0.110b	n.d.
